# Efficient Pause Extraction and Encode Strategy for Alzheimer’s Disease Detection Using Only Acoustic Features from Spontaneous Speech

**DOI:** 10.3390/brainsci13030477

**Published:** 2023-03-11

**Authors:** Jiamin Liu, Fan Fu, Liang Li, Junxiao Yu, Dacheng Zhong, Songsheng Zhu, Yuxuan Zhou, Bin Liu, Jianqing Li

**Affiliations:** 1 Jiangsu Province Engineering Research Center of Smart Wearable and Rehabilitation Devices, School of Biomedical Engineering and Informatics, Nanjing Medical University, Nanjing 211166, China; 2 The State Key Laboratory of Bioelectronics, School of Instrument Science and Engineering, Southeast University, Nanjing 211166, China

**Keywords:** Alzheimer’s disease detection, speech pause feature, machine learning, ensemble machine learning, statistical analysis

## Abstract

Clinical studies have shown that speech pauses can reflect the cognitive function differences between Alzheimer’s Disease (AD) and non-AD patients, while the value of pause information in AD detection has not been fully explored. Herein, we propose a speech pause feature extraction and encoding strategy for only acoustic-signal-based AD detection. First, a voice activity detection (VAD) method was constructed to detect pause/non-pause feature and encode it to binary pause sequences that are easier to calculate. Then, an ensemble machine-learning-based approach was proposed for the classification of AD from the participants’ spontaneous speech, based on the VAD Pause feature sequence and common acoustic feature sets (ComParE and eGeMAPS). The proposed pause feature sequence was verified in five machine-learning models. The validation data included two public challenge datasets (ADReSS and ADReSSo, English voice) and a local dataset (10 audio recordings containing five patients and five controls, Chinese voice). Results showed that the VAD Pause feature was more effective than common feature sets (ComParE: 6373 features and eGeMAPS: 88 features) for AD classification, and that the ensemble method improved the accuracy by more than 5% compared to several baseline methods (8% on the ADReSS dataset; 5.9% on the ADReSSo dataset). Moreover, the pause-sequence-based AD detection method could achieve 80% accuracy on the local dataset. Our study further demonstrated the potential of pause information in speech-based AD detection, and also contributed to a more accessible and general pause feature extraction and encoding method for AD detection.

## 1. Introduction

Alzheimer’s disease (AD) is an irreversible disease and there is little physicians can do when patients progress to advanced stages [1]. According to estimates from the World Alzheimer’s Disease Report 2019 and Alzheimer’s Disease International, there are over 50 million people living with dementia in the world, and the projected estimates for 2050 reach above 150 million [2]. Early detection holds great value for AD patients, as patients that are diagnosed early and seek treatment can be helped by numerous interventions to maintain their current state or delay cognitive decline [3].

Deterioration in speech and language production is among the first signs of the disease [4], and people with AD exhibit language impairment long before they are diagnosed [5]. In clinical practice, neuropsychological assessments are often used to initially screen for AD, evaluating their cognitive status through the patients’ manner of speaking and content [6]. However, this evaluation method relies on subjective assessment by human experts, which is difficult to quantify and meet the needs of AD patients for home testing.

Several researchers have focused on the use of spontaneous speech to detect AD. Yuan et al. [7] obtained the best accuracy of 89.6% using text-based features and acoustic features. Agbavor et al. [8] developed an end-to-end AD detection method with average area under the curve of 0.846. Although these methods have achieved good performance, they are still prone to voice-to-text transcriptional accuracy issues. For patients who are not native English speakers, and elderly people with dialect accents, the generalization ability of text-based feature AD detection methods may be limited.

Compared to text-based feature approaches, using only acoustic features allows for extracting direct information from the speech itself, which is more robust to the native language of the subject. Luz [9] used spontaneous speech data from the “cookie theft” picture description task in the Pitt database, extracted statistical and nominal acoustic features, and achieved a 68% accuracy using a Bayesian classifier. Eyben et al. [10] designed a unified feature set, Computational Paralinguistics ChallengE (ComParE), based on the features and lessons learned from the 2009–2012 challenge, which has general applicability in paralinguistic information extraction. Eyben et al. [11] proposed a standard acoustic parameter set, the extended Geneva minimalistic acoustic parameter set (eGeMAPS), such that the research results obtained in various areas of automatic speech analysis could be properly compared. Moreover, some researchers explored acoustic features for AD diagnosis from the perspective of digital signal processing, such as higher-order spectral features [12], fractal features [13], and wavelet-packet-based features [14].

Speech pauses can reflect the speaker’s cognitive function. Patients with neurodegenerative diseases have decreased cognitive function and often exhibit language impairment, such as frontotemporal lobar degeneration [15], Lewy body spectrum disorders [16], primary progressive aphasia [17] and AD [18]. Patients with AD experience disfluency, a high number of pauses, and repetition in speech, which is attributed to lexical retrieval difficulties due to cognitive decline [19]. Therefore, pauses are often used to analyze the speech and language of AD patients in order to assess their cognitive level [7,20,21].

Pauses mainly include filled and silent (unfilled) pauses. English has two common filled pauses: “uh” and “um” [7]. The term “silent pause” represents a temporal region in which a speaker does not utter a word, phrase, or sentence during spontaneous speech [22]. Researchers detected AD by calculating features such as the location, duration and frequency of the two types of pauses [23,24,25]. However, in some AD-related classification tasks, silent pauses performed better than filled pauses [23,26]. Moreover, silent pauses are easier to obtain from speech signals and more convenient for AD detection in daily conversation. In addition, silent pauses can be used to detect other acquired language disorders [27,28]. Therefore, this paper focuses on the study of silent pauses (hereafter, silent pauses are referred to as pauses).

Pauses have been shown to be effective in the detection of AD. Vincze et al. [23] acquired speech recordings by stimulating participants’ memory systems through three connected speech tasks, and used the PRAAT software for language analysis. The results show that the length, number, and rate of pauses differed between AD patients and healthy controls. Yuan et al. [7] likewise studied the variability of speech pauses between AD and healthy controls, classifying pauses into three discrete categories (under 0.5 s, 0.5–2 s, and over 2 s) based on their duration, with three types of punctuation (“,”, “.”, and “…”), marking the pauses in different groups and encoding them into word sequences to form pause-encoded transcripts. They used the ERNIE model for classification and achieved a high accuracy on the ADReSS test set. However, the approach remains a method of speech and language fusion, relying on speech transcription. Moreover, the discrete representation of pause features may not necessarily reflect all cognitive impairment information contained in the pause.

In order to provide a simple, widely available screening method for AD, this paper further explores the use of pauses in AD detection and proposes a voice activity detection (VAD)-based pausing feature recognition strategy, which can automatically generate pausing sequences in patients’ spontaneous speech. We compare the performance of the constructed pause features, with the public feature set on different machine-learning classifiers. The effectiveness of VAD Pause was verified on a local dataset. The main contributions of this study are listed as follows:

(1) We introduce an acoustic feature named the VAD Pause, extracted from speech signals on the newly shared ADReSS and ADReSSo English datasets, and the VAD Pause features are tested on a local Chinese dataset.

(2) A machine-learning-based ensemble approach using only acoustic features is proposed for AD classification.

(3) Statistical results revealed that VAD Pause features are higher in classification accuracy than the acoustic feature sets, and the ensemble method has higher classification value than the public feature-set-based method.

## 2. Materials and Methods

### 2.1. System Framework

**Figure 1:**
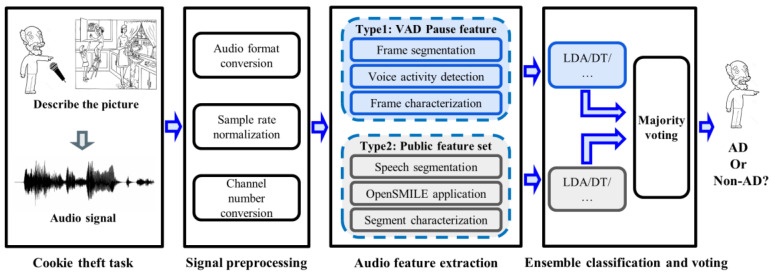
System framework for AD detection. LDA, linear discriminant analysis; DT, decision trees; AD, Alzheimer’s Disease.

The framework of the proposed method is shown in Figure 1. First, the participant was asked to perform a picture description task (“Cookie Theft” picture), during which their voice was recorded. Second, the speech signal was pre-processed to standardize it for next processing. Then, two types of features were extracted from speech signals. The first was the VAD-based pause feature proposed by the research, and the other was the feature from the common feature set. We compare the effectiveness of features on different classifiers. At the same time, in order to integrate the advantages of different types of features, we also designed a feature ensemble method based on the major voting strategy to obtain a better AD detection performance.

The following will be introduced in detail from dataset descriptions, information preprocessing, feature extraction, ensemble classification methods, and evaluation methods.

### 2.2. Dataset Descriptions

#### 2.2.1. Public Dataset

To evaluate the performance of our method, publicly available English audio datasets from references [29,30] were used for training and testing (ADReSS Challenge and ADReSSo Challenge). The challenges were organized by Reacher Saturnino Luz, Fasih Haider, and Sofia de la Fuente Garcia from the University of Edinburgh, and Davida Fromm and Brian MacWhinney of Carnegie Mellon University. They targeted a difficult automatic prediction problem of societal and medical significance, namely the detection of cognitive impairment and Alzheimer’s dementia. The competitions provide standard datasets of spontaneous speech, defining a shared task by which different spontaneous speech-based AD detection approaches can be compared.

The datasets consist of a set of recordings of picture descriptions produced by patients with an AD diagnosis and cognitively normal subjects, who were asked to describe the “Cookie Theft” picture from the Boston Diagnostic Aphasia Examination [31,32]. The ADReSS Challenge dataset consists of recordings of 156 participants, while the ADReSSo Challenge dataset contains speech clips of 237 participants, both of which have been balanced with respect to age and gender to eliminate potential confounding and bias. For the data used in the classification task, competitions were classified as both mild cognitive impairment (MCI) and the dementia stage as AD, with matched controls as non-AD.

The details of the ADReSS and the ADReSSo Challenge dataset are as follows:

1. Dataset ADReSS: 70% of the ADReSS2020 data were used as the training set, and 30% of the data were used as the test set;
2. Dataset ADReSSo: 70% of the ADReSSo2021 data were used as the training set, and 30% of the data were used as the test set. Table 1 shows the composition and distribution of the datasets we used.

#### 2.2.2. Local Dataset

A total of 10 audio signals (Chinses) were recorded in this study, including 5 in the AD group, and 5 in the control group (CN).

Patients were recruited from the Nanjing Brain Hospital in the Jiangsu Province, China. Cases assessed by experienced neurologists were based on the criteria for the clinical diagnosis of AD, which were established by the National Institute of Neurological and Communicative Disorders and Stroke (NINCDS) and the Alzheimer’s Disease and Related Disorders Association (ADRDA) workgroup in 1984 [33]. The Mini-Mental State Examination (MMSE) were adopted. A professional conducted the MMSE measurements prior to the picture description experiment.

The inclusion criteria varied according to the educational level of the patients. The inclusion criteria for illiterate patients were MMSE ≤ 17 and meeting the criteria for NINCDS-ADRDA. The inclusion criteria for primary school patients were MMSE ≤ 20 and meeting the criteria for NINCDS-ADRDA. The inclusion criteria for middle school and above patients were MMSE ≤ 24 and meeting the criteria for NINCDS-ADRDA. The exclusion criteria included: (1) speech impairment; (2) a history of severe stroke, extensive multiple cerebral infarction, critical cerebral infarction or severe white matter lesions; (3) previous psychiatric or psychological disorders; (4) other neurological disorders that cause cognitive impairment, e.g., Lewy body dementia, Parkinson’s disease, hydrocephalus, vascular cognitive; (5) other diseases that can cause dementia, such as severe anemia, thyroid disease, syphilis, HIV infection, etc.; (6) combined with serious heart, liver, kidney and other medical diseases, or combined with serious hypertension, diabetes and other complications; (7) unable to cooperate with the completion of cranial MRI or CT scan. The regional review board approved the use of human participants in this study. Patients signed written informed consent forms before participation. The study was approved by the ethics committee of The First Affiliated Hospital with Nanjing Medical University, Nanjing, China, in accordance with the Helsinki Declaration.

Referring to the challenges, participants were asked to describe the “Cookie Theft” picture. Speech signals were recorded using the SONY recording pen (ICD-TX660, Nanjing City, China). We collected 10 voice messages ranging in length from 25 s to 183 s. The audio recording information details are summarized in Table 2.

### 2.3. Preprocessing

Speech signal preprocessing was divided into three steps: audio format conversion, sample rate normalization, and channel number conversion. Based on the format conversion algorithm, the audio was unified into the ‘.wav’ format at first. Then, to normalize the audio, the audio sampling rate was unified to 44,100 Hz. Finally, we detected the number of channels of the audio, and converted them to mono audio.

### 2.4. Feature Extraction

Two kinds of acoustic features used in this study were introduced in detail. One was a pause feature based on the speech signal called VAD Pause, while the other was two commonly used acoustic feature sets, ComParE and eGeMAPS. We extracted the public feature sets for comparison with the VAD Pause, and fused them to propose an ensemble method for detecting AD.

#### 2.4.1. VAD Pause Feature

**Figure 2:**
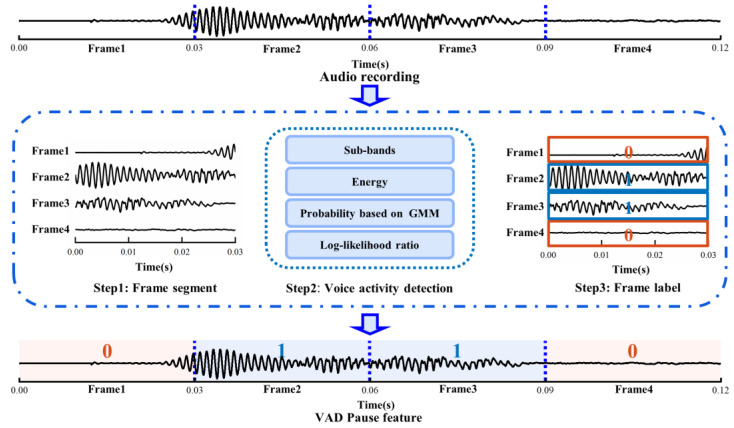
Schematic diagram of extracting VAD Pause. GMM, Gaussian mixture model.

Initially, we sectioned a voice recording into audio frames, and then used WebRTC VAD to detect each audio frame. Ultimately, a piece of audio datum was marked as the voiced and non-voiced frame according to the scale of audio frame, and this sequence was regarded as the pause feature of the speech. As shown in Figure 2, we set the frame duration to 0.03 s, such that the original 0.12 s of audio data turned into the VAD Pause feature, which was a sequence with four labels. Zero represents the non-voiced frame, and one represents the voiced frame.

The Google WebRTC VAD method was used to identify whether the audio frame was in a voiced or silent state. The detection procedure was as follows:

First, according to the correspondence between human speech pronunciation regulation and acoustic frequency, an audio frame was divided into six sub-bands: 80–250 Hz, 250–500 Hz, 500 Hz–1 kHz, 1–2, 2–3, 3–4 kHz. Next, the energy of the six sub-bands was calculated and denoted as *E*_1_, *E*_2_, *E*_3_, *E*_4_, *E*_5_, *E*_6_, and the total energy of the audio frame *E_t_*.

Then, if the total energy of the audio frame *E_t_* was greater than the energy threshold Tm, we proceeded to the following step:

1. For each sub-band, we made the assumptions that:

**H0.**  *the energy of the sub-band satisfied the Gaussian distribution of silent states.*

**H1.**  *the energy of the sub-band satisfied the Gaussian distribution of voiced states.*

2. For each sub-band, the probability that it belonged to the silent state was calculated based on the silent Gaussian mixture model (GMM):

(1) $$P\left(E_{i}|H0\right)=w_{s1}\frac{1}{\sqrt{2\pi }\sigma_{s1}}e^{\left(-\frac{{\left(E_{i}-\mu_{s1}\right)}^{2}}{2\sigma_{s1}^{2}}\right)}+w_{s2}\frac{1}{\sqrt{2\pi }\sigma_{s2}}e^{\left(-\frac{{\left(E_{i}-\mu_{s2}\right)}^{2}}{2\sigma_{s2}^{2}}\right)}$$

For each sub-band, the probability that it belonged to the voiced state was calculated based on the voiced GMM:

(2) $$P\left(E_{i}|H1\right)=w_{v1}\frac{1}{\sqrt{2\pi }\sigma_{v1}}e^{\left(-\frac{{\left(E_{i}-\mu_{v1}\right)}^{2}}{2\sigma_{v1}^{2}}\right)}+w_{v2}\frac{1}{\sqrt{2\pi }\sigma_{v2}}e^{\left(-\frac{{\left(E_{i}-\mu_{v2}\right)}^{2}}{2\sigma_{v2}^{2}}\right)},$$

where *E_i_* (*i* = 1, 2, …, 6) denotes the energy of the sub-band; *w_s*1*_*, *w_s*2*_* and *w_v*1*_*, *w_v*2*_* denote the weights for silent and voiced mixture Gaussian distributions, respectively; *μ_s*1*_*, *μ_s*2*_*, and *μ_v*1*_*, *μ_v*2*_* denote the respective means; *σ_s*1*_*, *σ_s*2*_*, and *σ_v*1*_*, *σ_v*2*_* denote the respective standard deviations.

3. The log-likelihood ratio *L_i_* for each sub-band and the total log-likelihood ratio *L_t_* were calculated according to the formula:

(3) $$L_{i}\left(E_{i}\right)=\log_{2}\left(\frac{P\left(E_{i}|H1\right)}{P\left(E_{i}|H0\right)}\right)$$

(4) $$L_{t}=\sum_{i=1}^{6}K_{i}L_{i}\left(E_{i}\right),$$

where *K_i_* denotes the weight each sub-band.

4. The thresholds *T_τ_* and *T_a_* were compared to determine whether the audio frame was audible or silent:

(5) $$F_{vad}=\{\begin{array}{l} 1,L_{i}>T_{\tau }\|L_{t}>T_{a}\\ 0,\;else \end{array},$$

where *T_τ_* denotes the threshold value of the sub-band log-likelihood ratio; *T_a_* denotes the threshold value of the total log-likelihood ratio.

#### 2.4.2. Common Acoustic Feature Sets

We used an open-source software package openSMILE toolkit, widely employed for emotion and affect recognition in speech [34], for acoustic feature set extraction of speech fragments. Following is a brief description of the two feature sets constructed in this manner:

ComParE: The ComParE 2013 [10] feature set includes energy, spectral, Mel-Frequency Cepstral Coefficients (MFCC), and logarithmic harmonic-to-noise ratio, voice quality features, Viterbi smoothing for F0, spectral harmonicity, and psychoacoustic spectral sharpness. Furthermore, statistical functions were applied to these features, bringing the total to 6373 features for every speech segment.

eGeMAPS: The eGeMAPS [11] feature set contains the F0 semitone, jitter, shimmer, loudness, spectral flux, MFCC, F1, F2, F3, alpha ratio, Hammarberg index, and slope V0 features. Statistical functionals were also computed, for a total of 88 features per speech segment.

### 2.5. Ensemble Classification and Voting

**Figure 3:**
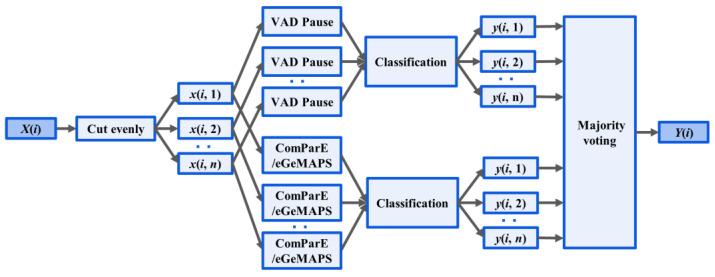
Ensemble Method: *X*(*i*), the audio recording, was divided into n segments *x*(*i, n*). Acoustic feature extraction (VAD Pause, ComParE and eGeMAPS) was performed at the segment level. The output of classification for the *nth* segment of the *ith* recording is denoted *y*(*i, n*). *Y*(*i*) outputs the majority voting for classification.

A novel fusion method was introduced based on the mentioned features and classifiers, as illustrated in Figure 3. Two-step classification experiments were conducted to detect AD. First, the signal was segmented into four s-long sequences after preprocessing and use of the segment-level classification, where classifiers were trained and tested to predict whether a speech segment was uttered by an AD or non-AD patient. Then, we calculated the majority vote from the segment-level results of classifiers, and returned a class label for each subject.

The classify model included five classic machine-learning methods, namely: linear discriminant analysis (LDA), decision trees (DT), k-nearest neighbor (KNN), support vector machines (SVM with a linear kernel and a sequential minimal optimization solver), and tree bagger (TB).

In order to focus on the effect of speech features and avoid the interference of classifier factors, the traditional machine-learning model, with relatively mature research and strong interpretability, was used as the classifier of this paper. When the validity of the proposed pause feature sequence was determined, such sequence could be directly used as input for training the depth neural network classifier. It could also be further fused with other semantic-based AD detection features to obtain better classification performance.

### 2.6. Evaluation

#### 2.6.1. Classification Metrics

Depending on whether the predicted label matched with the true label, the outputs of a binary classification algorithm fell into one of the four categories: true positives (*TP*), false positives (*FP*), false negatives (*FN*), and true negatives (*TN*). The classification metrics are defined as follows:

(6) $$Accuracy=\frac{TP+TN}{TP+TN+FP+FN}$$

(7) $$Precision=\frac{TP}{TP+FP}$$

(8) $$Recall=\frac{TP}{TP+FN}$$

(9) $$F1score=2\frac{\left(Precision\right)\left(Recall\right)}{Precision+Recall}$$

#### 2.6.2. Statistical Analysis

To explore whether the feature was truly effective from the perspective of data analysis, the effects of classifiers and features on classification accuracy were tested with a two-way ANOVA (feature (ComParE, eGeMAPS, VAD Pause, ensemble) and classifier (LDA, DT, KNN, SVM, TB) as factors. If there was no interaction between the factors, further multivariate analysis could be performed to analyze the inter-group differences of a single factor, while if the interaction between the factors appeared significant, multiple analysis was further conducted to determine which level of combination of classifiers and features had the highest average classification accuracy.

## 3. Results

The results are presented in four parts. First, an intuitive comparison of pause features in AD and non-AD populations is provided. Second, quantitative comparison results of our method are shown in two datasets. Then, a statistical analysis of the classification methods was conducted to demonstrate the validity of the proposed pause feature and the ensemble method. Finally, the VAD Pause features were tested on our own collected Chinese dataset.

### 3.1. Comparison of VAD Pauses in AD and Non-AD Subjects

**Figure 4:**
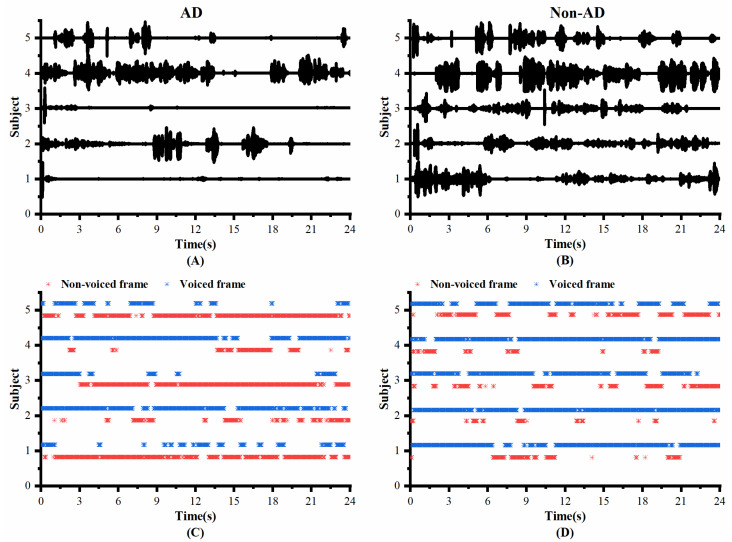
Contrast display of representative signals from AD and non-AD subjects: (**A**) raw audio recordings of AD; (**B**) raw audio recordings of non-AD; (**C**) VAD Pause features of AD; (**D**) VAD Pause features of non-AD. The blue asterisks represent voiced frames, whereas red represent non-voiced frames in the figure of pause features.

To investigate whether the speech of the AD and controls differed in pauses, we visualized the original speech recordings and VAD Pause features to analyze and confirm the difference from a visual point of view. We selected ten audio clips (half of the speakers were AD subjects, and half were non-AD) of about 1 min length from the test set of the ADReSS2020 dataset. Then, we intercepted the first 24 s of data and extracted their VAD Pause features from them. Figure 4 shows the raw voice waveform (A,B) and VAD Pause features of the voice recordings (C,D).

Figure 4 shows some differences in the speech between the AD and non-AD subjects. From the comparison, the subjects with AD had more pauses in every group, and exhibited poor speech coherence compared to healthy controls. The speech recordings of healthy controls contained more voiced frames. Although pauses were present, the speech was more continuous than in the AD group.

### 3.2. Quantitative Results in Classic Machine-Learning Methods

**Figure 5:**
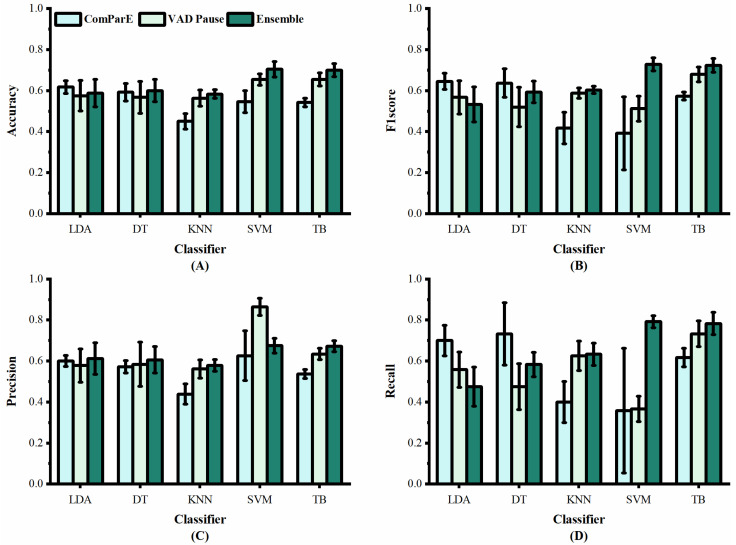
Performance of features from our study and [29] on dataset ADReSS: (**A**) accuracy; (**B**) F1 score; (**C**) precision; (**D**) recall.

**Figure 6:**
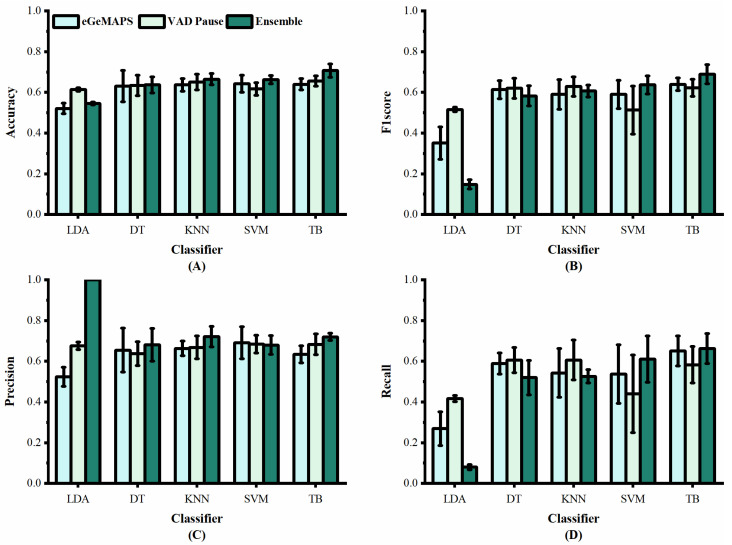
Performance of features from our study and [30] on dataset ADReSSo: (**A**) accuracy; (**B**) F1 score; (**C**) precision; (**D**) recall.

We applied the VAD Pause to five machine-learning methods employed previously by [29,30], namely LDA, DT, KNN, SVM, and TB, for AD and non-AD classification. The classification results of all methods on the datasets ADReSS and ADReSSo, are reported in Figure 5 and Figure 6, respectively. For a fair comparison, the ComParE with the best effect used in the literature [29] was selected and tested on the dataset ADReSS, while the eGeMAPS used in the literature [30] was selected and tested on the dataset ADReSSo. All classifiers used the same parameters as those in the literature, and each classification was performed with five runs and shuffling the data order. The average results of the operations are shown in the figures.

Figure 5 shows the classification results of the proposed VAD Pause feature and ComParE, using five machine-learning methods on dataset ADReSS. It can be observed that the VAD Pause had a better classification effect and smaller error bars than the ComParE on three machine-learning methods KNN, SVM, and TB. Our VAD Pause with the TB had the best effect, reaching 65.4% accuracy, 67.9% F1 score, 63.4% precision, and 73.3% recall. This indicates better performance than the acoustic feature-based baseline accuracy of 62.5% obtained by [29].

Figure 6 shows the classification results of the proposed VAD Pause feature and eGeMAPS, using five machine-learning methods on the dataset ADReSSo. It can be observed that the VAD Pause feature proposed in this study had a better classification performance and smaller error bars than the eGeMAPS on the same dataset using LDA, KNN, DT, and TB. Our VAD Pause with the TB had the best effect, reaching 65.6% accuracy, 62.3% F1 score, 68.3% precision, and 58.3% recall. Thus, it performed better than the acoustic feature-based baseline accuracy of 64.8% obtained by [30].

Although the VAD Pause gains advantages compared to ComParE and the eGeMAPS, a method that can improve the classification results is required. Thus, we introduced the ensemble procedure. We observed that the TB performs best (in terms of accuracy and stability), and the results are shown in Table 3. Our findings indicate that combining the public feature set (ComParE and eGeMAPS) with the VAD Pause improves the classification results. We further observe the variance from Figure 5 and Figure 6. The proposed ensemble method (dark green) improves the mean and reduces variance overestimates.

The classification performance of the presented ensemble method was also compared against other AD detection approaches on the test sets in the literature, shown in Table 3. Our proposed ensemble method achieved the best performance among methods without the deep-learning (DL) model.

### 3.3. Statistical Analysis of Classification Methods

**Figure 7:**
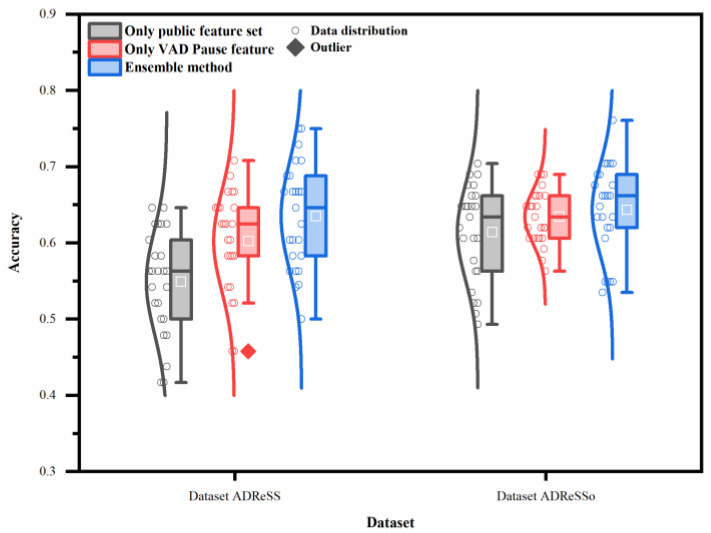
Performance of different features or ensemble method on two datasets (datasets ADReSS and ADReSSo), using five machine-learning classifiers.

To verify whether our method achieves significant improvement, statistical analysis was used to compare the classification results. Figure 7 illustrates the overall effect of different features or the ensemble method, using five machine-learning classifiers on the two datasets. In contrast to the public feature set, the proposed feature and method have a higher average recognition rate, with less recognition dispersion in testing. Thus, the proposed feature and method exhibit better generalization ability.

The two-way ANOVA reveals that both classifiers and features contribute to the differences noted in the classification accuracy (*p* < 0.05). The interaction between them is also very significant on the datasets ADReSS and ADReSSo (*p* < 0.05).

**Figure 8:**
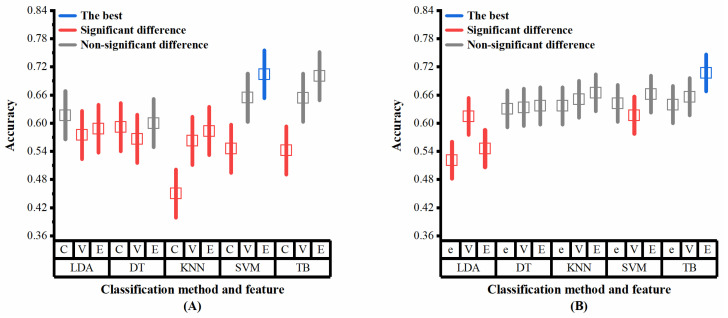
Multiple analysis results (classifiers and features as factors) on the two datasets: (**A**) dataset ADReSS; (**B**) dataset ADReSSo. C, ComParE; V, VAD Pause; E, ensemble; e, eGeMAPS. Blue lines represent the optimal performance, red lines represent significant difference from the optimal performance, gray lines represent non-significant difference from the optimal performance.

Multiple analysis is further conducted to determine the level of combination of classifiers and features that had the highest average classification accuracy, as the interaction between classifiers and features appeared significant on the datasets ADReSS and ADReSSo. The results of the multiple analysis (Figure 8A,B) show that slightly different results are obtained from different datasets. These combinations achieved the best results on both sets of data: the ensemble method with DT, the ensemble method with SVM, the VAD Pause feature with TB, and the ensemble method with TB. The simple effects analyses confirmed that the proposed ensemble method and VAD Pause-based method have significantly higher classification accuracies than the public feature set-based method, which is consistent with our results in Section 3.2.

### 3.4. Experimental Results on a Local Dataset

In aiming to test the effectiveness of VAD Pause features in practical applications, we initially collected ten Chinese speech recordings for experimentation. The ADReSS and ADReSSo English datasets were mixed to serve as the training set after the overlapping data were removed. Then, the five classifiers were fed separately for training, and finally the classification results were tested separately on the local Chinese dataset.

**Figure 9:**
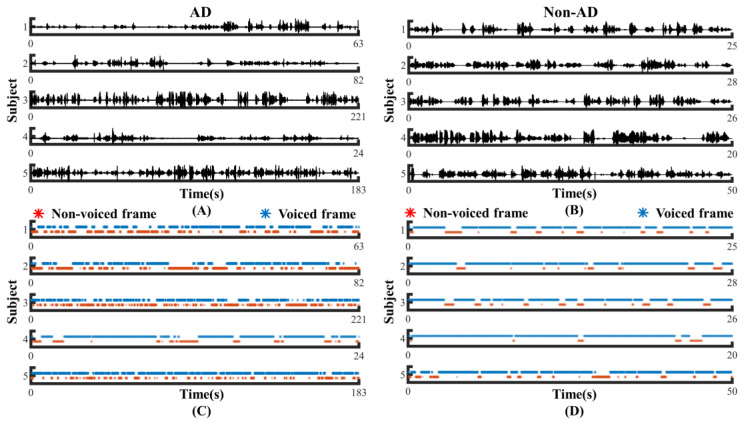
Contrast display of clinical data from AD and non-AD subjects: (**A**) raw audio recordings of AD; (**B**) raw audio recordings of non-AD; (**C**) VAD Pause features of AD; (**D**) VAD Pause features of non-AD. The blue asterisks represent voiced frames, whereas the red asterisks represent non-voiced frames in the figure of pause features.

As shown in Table 4, the VAD Pause feature obtained good classification results on the local Chinese dataset. The classification was basically correct for all AD patients, but there was potential for improvement in the classification of healthy individuals. To further explore the causes of classification errors, the raw waveforms and VAD Pause features of the local Chinese speech dataset are shown in Figure 9.

As seen in Figure 9, there were more and longer pauses in the speech of AD patients compared to healthy people in the local dataset, which is consistent with our previous results discussed in Figure 4. It is normal that 1, 3, and 5, which have a relatively high number of pauses in healthy people, have the possibility of being misclassified, which may be related to the existence of pauses in or between utterances in Chinese itself. Additionally, the native language of the participating subjects in the local dataset was Chinese with interspersed dialects, while the public dataset was English. Our proposed features have significant discriminative power on the test subjects of different languages, which shows the advantage of AD recognition based on speech features only.

## 4. Discussion

In this paper, we tried to mine the AD symptom information contained in the speech signal itself from the pause perspective, and proposed a VAD-Pause-based method for AD detection that can be applied to easily and conveniently screen for AD in the future. The method was tested for AD classification on the English public datasets and our own local Chinese dataset. We explored the effect of applying the VAD Pause and machine-learning methods, and a novel ensemble method, as well as possible independence/interdependence of their association with the AD classification. The results confirm that AD subjects use more pauses than healthy controls, and that the VAD Pause and ensemble method have higher classification values and better generalization ability than public acoustic feature sets.

The effectiveness and generalization ability of the VAD Pause for AD detection constitute the most intriguing results. VAD Pause performance can be attributed to the difference in pauses in speech between the two groups. Previous studies reported that in speech production, disfluencies, such as hesitations and speech errors, are correlated with cognitive functions, such as cognitive load, arousal, and working memory [45,46]. Semantic verbal fluency and phonological verbal fluency tests are widely used in the diagnosis of AD, and they are reliable indicators of language deterioration in the early detection of AD [47]. Another study suggested that AD patients require more effort to speak than healthy individuals: namely, patients speak more slowly with longer pauses [48]. Yuan et al. [7] studied the function of pauses from the perspective of manual transcription, and concluded that AD subjects used more and longer pauses than healthy people. All of these results seem to suggest that pauses have a potential status in the distinction between the AD and non-AD. Furthermore, the VAD Pause may be a valid feature that represents pauses well. Figure 4 and Figure 9 show that generalizing the difference between the two for original recordings, which hold a significant amount of information, is not a trivial task, whereas the VAD Pause feature proposed in this study shows the difference visually.

Notably, our result is representative of the performance of AD detection when using only audio recordings, without transcription. Several reasons account for the advantages of this practice. First, it is more practical to detect AD using speech directly. When a physician conducts a clinical interview, they usually evaluate the patient directly based on their voice, rather than transcribing the words. When the program detects AD directly via speech, it takes less time than when needing to transcribe it into text. Moreover, in this study, we use pauses as detection features, while transcription had an effect on the detection of pauses. A prior study showed that transcription errors impacted findings related to the usefulness of prosodic features in parsing [49]. Several state-of-the-art approaches rely on this manually generated text for feature extraction, and their performance may vary depending on whether the transcription is automated [3]. If we use speech directly for feature extraction and AD detection, the influence of transcription can be avoided, and the error generated in the process may be reduced.

Comparisons with other methods using the same data attract particular interest. Table 3 shows that methods using acoustic features for AD detection can be divided into two categories: those that use DL and those that do not. Our ensemble method achieves the best results among methods that do not rely on DL [29,30,39,44]. In the dataset ADReSS, the pre-trained VGGish model and Uni-CRNN are used in the method proposed by [35]. An accuracy of 72.9% was achieved with this method. In addition, among the DL-related approaches, some studies use neural networks as classifiers, while others focus on using them to extract acoustic embeddings. Cummins et al. [36] and Rohanian et al. [37] combined public acoustic features with neural networks and achieved an accuracy of 70.8 and 66.6%, respectively. Acoustic embeddings as speech features started to attract the attention of numerous researchers, and have gained good performance in AD detection [38,40,41,42,43]. There appears to be a trade-off in accuracy and convenience. Methods using DL are more accurate, but methods without DL are more convenient. A better approach could involve using VAD Pause and acoustic embeddings to represent speech information. Due to time cost and practicality, we will further explore this idea in future.

We showed that both the features and classifiers used in this study contribute to the classification performance, as the results of the two-way ANOVA were significant in all two datasets. Moreover, in the datasets ADReSS and ADReSSo, the factor contribution to the highest average classification accuracy was intertwined. Statistically, the ensemble method with DT, the ensemble method with SVM, the VAD Pause feature with TB, and the ensemble method with TB have similarly high accuracies. Based on the analysis of these results, we recommend the ensemble method with TB, as it achieves stable and high accuracy, while several other methods are likewise available (ensemble method with DT, ensemble method with SVM, VAD Pause feature with TB). Additionally, we did not explore the effect of using DL networks for classification. Yet, usually DL classification requires a significant amount of data and time, such that it is necessary to optimize and improve the algorithm, which will be a future direction of our study.

Silent pauses have been implemented to other populations beyond AD. Some researchers have investigated the use of silent pause features for disease detection. Mignard et al. [50] investigated fluency disorders in Parkinson’s patients by the pause ratio. Potagas et al. [51] used speech rate, articulation rate, pause frequency, and pause duration as analytical indicators, and the results showed that silent pauses can be used as complementary biomarkers for PPA. Imre et al. [52] conducted a study on the temporal speech characteristics of elderly patients with type 2 diabetes (T2DM). The healthy cognition participants in the T2DM group showed higher duration rate of silent pause and total pause, and a higher average duration of silent pauses and total pauses compared to the group without T2DM group. These methods were mainly carried out based on the calculation of the frequency and duration of silent pauses, etc. Compared to previous publications, the method we propose to encode speech into a sequence of pauses can characterize the temporal sequence of pauses in speech more accurately, while the data processing is simpler and easy to implement and repeat.

Spontaneous speech analysis plays an important role in the study of acquired language disorders. Ditthapron et al. [27] used smartphones to passively capture changes in acoustic characteristics of spontaneous speech for continuous traumatic brain injury monitoring. Spontaneous speech can also be used for research on depression [53] and aphasia [28,54]. Thus, our proposed spontaneous speech-based approach has the potential to be used in other clinical populations with acquired language disorders. In future work, we consider investigating the feasibility of applying this method to other populations.

## 5. Conclusions

We proposed a pause/non-pause feature sequence (VAD Pause) encoded using only speech, and investigated its effectiveness when applied to distinguish AD patients from healthy subjects. Its classification effect was tested on both public datasets and a local dataset. We further introduced an ensemble method for AD classification from spontaneous speech and investigated the impact of features and classifiers on the results in detail, further demonstrating the superiority of the ensemble method through a comprehensive comparison of classification results of different datasets (two English datasets and one Chinese dataset). The results of the study suggest that our method can be partly immune to AD detection errors due to the language environment, which is of greater value for the widespread screening of AD. In future work, the proposed AD screening method will be considered extended to the detection of other acquired speech disorders, with appropriate modifications. We will also collect more local data to further demonstrate the reliability and potential of our proposed method.

## Figures and Tables

**Table 1:** Data composition of English public datasets.

	Dataset	AD	Non-AD	Total
Dataset ADReSS	Training	54	54	108
	Test	24	24	48
	Total	78	78	156
Dataset ADReSSo	Training	87	79	166
	Test	35	36	71
	Total	122	115	237

**Table 2:** Audio information of local Chinese dataset.

	Sample	MMSE	Age	Gender
CN	1	26	77	male
	2	27	73	male
	3	27	69	female
	4	29	74	male
	5	29	74	male
AD	6	11	70	female
	7	18	60	female
	8	21	84	female
	9	17	80	female
	10	11	79	female

CN, Control Group; AD, Alzheimer’s Disease Group; MMSE, Mini-Mental State Examination.

**Table 3:** Accuracy (ACC) comparison of the ensemble method with other approaches, using only acoustic features on the test sets in the literature.

Data	Study	Extracted Features	Classifiers	ACC (%)	DL Used
Dataset ADReSS	Koo et al. [35]	VGGish	Uni-CRNN	72.9	Yes
	Cummins et al. [36]	Log-Mel spectrograms	SiameseNet	70.8	Yes
	Rohanian et al. [37]	COVAREP	LSTM	66.6	Yes
	Pappagari et al. [38]	X-vectors and silence features	PLDA	66.7	Yes
	Edwards et al. [39]	ComParE	LDA	60.4	No
	Luz et al. (Baseline) [29]	ComParE	LDA	62.5	No
	**Our method**	**VAD Pause feature and ComParE**	**TB**	**70.0**	**No**
Dataset ADReSSo	Balagopalan et al. [40]	Conventional acoustic features and wav2vec2.0 pre-trained acoustic embeddings	SVM	67.6	Yes
	Pan et al. [41]	Wav2vec2.0 pre-trained acoustic embeddings	TB	74.7	Yes
	Pérez-Toro et al. [42]	X-vectors and dominance embeddings	RBF-SVM	67.6	Yes
	Pappagari et al. [43]	X-vectors, x-vectors (250ms) and encoder–decoder ASR embeddings	LR	74.7	Yes
	Chen et al. [44]	MFCC, GeMAPS, eGeMAPS, ComParE and IS10-Paralinguistics	LR	67.6	No
	Luz et al. (Baseline) [30]	eGeMAPS	SVM	64.8	No
	**Our method**	**VAD Pause feature and eGeMAPS**	**TB**	**70.7**	**No**

Uni-CRNN, unimodal convolutional recurrent neural network; DL, deep learning; SiameseNet, Siamese network; LSTM, long short-term memory; PLDA, probabilistic linear discriminant analysis; LDA, linear discriminant analysis; TB, tree bagger; SVM, support vector machine; RBF-SVM, radial basis function-support vector machine; ASR, automatic speech recognition; LR, logistic regression; MFCC, Mel-frequency cepstral coefficients.

**Table 4:** Performance of the VAD Pause feature on a local dataset, using five machine-learning classifiers.

Ture	Sample	LDA	DT	KNN	SVM	TB
CN (0)	1	1	0	0	1	1
	2	0	0	0	0	0
	3	1	1	0	1	1
	4	0	0	0	0	0
	5	1	0	1	0	1
AD (1)	6	1	1	1	1	1
	7	1	1	1	1	1
	8	1	1	1	1	1
	9	1	1	0	1	1
	10	1	0	1	1	1
	**Accuracy (%)**	**70**	**80**	**80**	**80**	**70**

CN, control group; AD, Alzheimer’s Disease group; LDA, linear discriminant analysis; DT, decision trees; KNN, nearest neighbor; SVM, support vector machine; TB, tree bagger.

## Data Availability

The local datasets analyzed during the current study are not publicly available due to patient privacy, but are available from the corresponding author on reasonable request. Public challenge datasets can be found at: https://dementia.talkbank.org/ADReSS-2020/ and https://dementia.talkbank.org/ADReSS-2021/ (accessed on 8 October 2021).
